# Advancements in sarcopenia diagnosis: from imaging techniques to non-radiation assessments

**DOI:** 10.3389/fmedt.2024.1467155

**Published:** 2024-10-09

**Authors:** Salvatore Lavalle, Rosa Scapaticci, Edoardo Masiello, Carmelo Messina, Alberto Aliprandi, Valerio Mario Salerno, Arcangelo Russo, Francesco Pegreffi

**Affiliations:** ^1^ Department of Medicine and Surgery, Kore University of Enna, Enna, Italy; ^2^ Institute for the Electromagnetic Sensing of the Environment, National Research Council of Italy, Naples, Italy; ^3^ Department of Radiology, IRCCS San Raffaele Scientific Institute, Milan, Italy; ^4^ Department of Radiology, IRCCS Istituto Ortopedico Galeazzi, Milan, Italy; ^5^ Department of Biomedical Sciences for Health, University of Milan, Milan, Italy; ^6^ Department of Radiology, Zucchi Clinical Institutes, Monza, Italy

**Keywords:** sarcopenia, dual-energy x-ray absorptiometry, computed tomography, ultrasound imaging, magnetic resonance imaging, bioelectrical impedance analysis, quantitative parameters, non-ionizing radiation

## Abstract

Sarcopenia is a prevalent condition with significant clinical implications, and it is expected to escalate globally, demanding for effective diagnostic strategies, possibly at an early stage of the disease. Imaging techniques play a pivotal role in comprehensively evaluating sarcopenia, offering insights into both muscle quantity and quality. Among all the imaging techniques currently used for the diagnosis and follow up of sarcopenia, it is possible to distinguish two classes: Rx based techniques, using ionizing radiations, and non-invasive techniques, which are based on the use of safe and low risk diagnostic procedures. Dual-energy x-ray Absorptiometry and Computed Tomography, while widely utilized, entail radiation exposure concerns. Ultrasound imaging offers portability, real-time imaging, and absence of ionizing radiation, making it a promising tool Magnetic Resonance Imaging, particularly T1-weighted and Dixon sequences, provides cross- sectional and high-resolution images and fat-water separation capabilities, facilitating precise sarcopenia quantification. Bioelectrical Impedance Analysis (BIA), a non-invasive technique, estimates body composition, including muscle mass, albeit influenced by hydration status. Standardized protocols, such as those proposed by the Sarcopenia through Ultrasound (SARCUS) Working Group, are imperative for ensuring consistency across assessments. Future research should focus on refining these techniques and harnessing the potential of radiomics and artificial intelligence to enhance diagnostic accuracy and prognostic capabilities in sarcopenia.

## 1 Introduction

Sarcopenia is recognized as a prevalent and clinically relevant condition, particularly in aging populations (1). Depending on the criteria used for diagnosis and the screened population, prevalence rates range from 0.2% to 86.5% (2). Research findings from systematic reviews highlight variations in prevalence, with higher rates observed among individuals residing in long-term residential care settings (14%–33%) compared to those leading an active lifestyle (1%–29%) (1). Projections indicate a substantial increase in sarcopenia's worldwide prevalence, expected to rise from 50 million individuals in 2010 to approximately 200 million by 2050 (3). In addition to muscle weakness and loss of muscle mass, sarcopenia may manifest with various clinical symptoms affecting overall physical function and quality of life. These symptoms can include reduced mobility, balance problems, increased risk of falls and fractures, difficulty performing activities of daily living, and an overall decline in functional independence (4, 5). Sarcopenia can also contribute to metabolic changes, such as insulin resistance, and may exacerbate existing health conditions like diabetes, cardiovascular disease, and obesity (6). Sarcopenia arises from a complex interplay of factors involving both age-related physiological changes and various pathological mechanisms (7). The etiopathogenesis of sarcopenia is multifactorial and involves alterations in muscle protein turnover, hormonal changes (such as reduced levels of testosterone, growth hormone, and insulin-like growth factor-1, chronic inflammation, oxidative stress, mitochondrial dysfunction, and neuronal deficits (8–10). These factors contribute to an imbalance between muscle protein synthesis and degradation, ultimately leading to muscle wasting and weakness (11). Physiologically, sarcopenia is characterized by structural and functional changes within skeletal muscle, including fiber atrophy, decreased muscle fiber number and size, increased intramuscular fat deposition, impaired muscle contractility, and reduced muscle metabolic activity (12, 13). Histologically, sarcopenia is associated with alterations in muscle fiber composition, such as a shift from type II (fast-twitch) to type I (slow-twitch) muscle fibers, as well as increased fibrosis and infiltration of adipose tissue within muscle (14, 15). Biochemically, sarcopenia is marked by dysregulation of various signaling pathways involved in muscle protein synthesis and degradation, including the mTOR (mechanistic target of rapamycin) pathway, the ubiquitin-proteasome system, and autophagy (16–18). Although the diagnosis of sarcopenia is currently mainly performed via clinical examination, evaluating parameters like muscle strength and muscle mass, imaging diagnostic techniques can play a key role in the comprehensive evaluation of sarcopenia, providing valuable insights into both the quantitative and qualitative aspects of muscle structure, as well as providing information for the early detection of the disease (19, 20). Imaging techniques can be gathered into two main categories: invasive and non-invasive ones. The first category includes techniques and procedures that pose some risks for patients and clinical operators. The main representatives of this class of imaging techniques are those using ionizing radiations (x-rays) as a means to inspect the human body. Among the techniques used in sarcopenia evaluation, dual-energy x-ray Absorptiometry (DEXA) employs x-ray beams to measure bone density and soft tissue composition, including muscle mass (21). However, it cannot quantify intramuscular adipose, impacting muscle quality estimation, and factors like body thickness and hydration status can influence results. DEXA may overestimate muscle mass in cases of fluid accumulation and may provide inaccurate assessments of fat and muscle mass in obese individuals (22). Computed Tomography (CT) is also commonly employed in both oncological and non-oncological diagnostic settings and is increasingly utilized for screening sarcopenia due to its efficacy in assessing muscle mass and quality. CT is considered a gold standard for body composition analysis, particularly in nutritionally vulnerable patients (23). Its prevalence in medical practice is rising, with increased usage projected globally. CT's versatility and increased usage are attributed to its effectiveness in diagnosing various acute and chronic illnesses associated with aging. However, a significant drawback of this technique is radiation exposure, limiting its role as a screening tool for sarcopenia (24). Non-invasive imaging technologies, on the other hand, are based on the use of non-ionizing radiations, which can be safely adopted, even for repeated examinations, without increasing the carcinogenic risk to patients. This work aims to explore the significance of non-radiation techniques in the quantification of sarcopenia, shedding light on their unique capabilities, advantages, and potential applications in advancing our understanding of musculoskeletal health in aging populations. In particular, ultrasounds, magnetic resonance imaging (MRI)-based techniques, and bio-impedance assessment (BIA) will be described in detail, and a critical review of their use and application in sarcopenia diagnosis and evaluation will be reported.

## 2 Ultrasounds

### 2.1 Principles of ultrasound imaging in sarcopenia assessment

Ultrasound (US) imaging utilizes high-frequency sound waves to produce real-time images of soft tissues, including muscles. In the context of sarcopenia assessment, US involves the measurement of muscle size, architecture, and composition. The procedure typically involves positioning the subject in a supine position with legs fully extended. A linear probe, usually operating at frequencies ranging from 5 to 12 MHz, is commonly employed, with the anterior compartment of the thigh being one of the most frequently targeted sites (25). The imaging process involves the transmission of sound waves into the body, which then bounce back (echo) from the tissues, producing detailed images that can be analyzed for quantitative and qualitative assessments of muscle health and integrity.

### 2.2 Quantitative parameters

Muscle size is typically quantified by assessing muscle thickness (MT) and cross-sectional area (CSA), which have demonstrated strong correlations with other primary methods for studying muscle quantity, such as MRI, CT, and DXA (26, 27). MT refers to the depth or thickness of a muscle (28). In individuals with sarcopenia, muscle thickness may decrease due to the loss of muscle fibers and atrophy of existing muscle tissue (29). This reduction in muscle thickness can be observed in various muscle groups throughout the body, including the quadriceps, biceps, and calf muscles (30). CSA, in the context of muscle physiology, refers to the area of a muscle when viewed in a cross-section (31). It provides valuable information about the size and mass of the muscle. In sarcopenia, a reduction in CSA is linked to the decline in muscle strength and physical performance (32). Qualitative descriptions of muscle characteristics include parameters such as echo intensity (EI), pennation angle (PA), physiological cross-sectional area (PCSA), and fascicle length (FL) (33) (Table 1). EI is used to assess the quality or composition of tissues, including muscles. It refers to the brightness or darkness of the echoes produced by tissues in response to ultrasound waves (34). Higher EI values typically indicate greater amounts of intramuscular fat or fibrosis, whereas lower values suggest healthier muscle tissue. EI is quantified by analyzing the grayscale levels of the ultrasound images (35, 36). PA represents the angle between the muscle fibers and the tendon to which they attach (37). A larger PA indicates that the muscle fibers are oriented more obliquely relative to the tendon, suggesting a greater PCSA and the potential for greater force generation (38). Muscles with larger PA typically have more fibers per unit area, allowing for increased force production (39, 40). FL refers to the length of individual muscle fibers within a muscle. Longer fascicles generally allow for greater excursion and range of motion, which can be advantageous for activities requiring large muscle movements. Reduction in FL may be indicative of muscle atrophy or degeneration in individuals with sarcopenia (41, 42). Alternative US techniques, such as sonoelastography and contrast-enhanced US, have been suggested as potential tools for evaluating different aspects of muscle health in sarcopenia. Sonoelastography assesses changes in muscle stiffness, providing insights into tissue composition and integrity by measuring the elasticity of muscles (43). Contrast-enhanced US evaluates the microvasculature within muscles, which may be compromised in sarcopenia due to reduced blood flow and perfusion (44). However, research on these techniques in the context of sarcopenia is still limited, and further studies are needed to validate their utility and establish their role in clinical practice (21).

**Table 1:** Quantitative parameters in US.

Quantitative parameter	Description
Muscle Thickness (MT)	Depth or thickness of a muscle, often decreases in individuals with sarcopenia.
Cross-Sectional Area (CSA)	Area of a muscle when viewed in cross-section, indicative of muscle size and mass.
Echo Intensity (EI)	Brightness or darkness of echoes produced by tissues, reflecting muscle composition.
Pennation Angle (PA)	Angle formed between muscle fibers and the tendon to which they attach, influencing muscle force.
Physiological Cross-Sectional Area (PCSA)	Area of a muscle perpendicular to its fibers, correlates with muscle force generation.
Fascicle Length (FL)	Length of individual muscle fibers within a muscle, influencing mechanical properties and function.

These quantitative parameters provide valuable insights into various aspects of muscle health and composition, aiding in the assessment of sarcopenia through ultrasound imaging.

### 2.3 Clinical application

US has shown promise in various applications related to sarcopenia assessment (Table 2). In clinical practice, ultrasound imaging can be used for screening, diagnosis, and monitoring of sarcopenia (45). However, the proficiency and expertise of operators can significantly influence the accuracy and reliability of ultrasound measurements (46). A recent meta-analysis conducted by Wang et al. highlighted substantial methodological variation across studies, including differences in the type of ultrasonographic probe used, the positioning of the probe on the body, the specific side of the body measured, the posture of the subjects during measurements, and the specific anatomical points targeted for measurement (47). These methodological disparities underscore the need for standardized protocols and guidelines to ensure consistency and comparability across studies in the field of ultrasound-based assessment of sarcopenia. The Sarcopenia through Ultrasound (SARCUS) Working Group has recently issued two consensuses aimed at standardizing ultrasound measurements for assessing appendicular muscles. However, due to a lack of sufficient evidence, the SARCUS consensuses have not yet established specific cutoff points for US parameters across different muscle groups to diagnose sarcopenia (48). More research is needed to establish ideal cutoffs for ultrasound parameters across diverse populations, enhancing the accuracy and utility of ultrasound in sarcopenia assessment.

**Table 2:** Overview of the role of ultrasound in assessing sarcopenia.

Aspect	Description
Principles of ultrasound	Utilizes high-frequency sound waves to produce real-time images of soft tissues, including muscles. In sarcopenia assessment, it measures muscle size, architecture, and composition.
Quantitative parameters	Muscle size is quantified by assessing muscle thickness (MT) and cross-sectional area (CSA), which correlate strongly with MRI, CT, and DXA. CSA specifically indicates the muscle's area in a cross-section. Other parameters include echo intensity (EI), pennation angle (PA), physiological cross-sectional area (PCSA), and fascicle length (FL).
Other US techniques	Sono-elastography assesses muscle stiffness, while contrast-enhanced US evaluates microvasculature changes associated with sarcopenia. Limited data is available on the utility of these technique in sarcopenia.
Clinical application	US can be used for sarcopenia screening, diagnosis, and monitoring in clinical practice. Operator proficiency significantly affects measurement accuracy. Standardized protocols, such as those by the SARCUS Working Group, aim to ensure consistency and comparability in ultrasound-based assessment.

## 3 Magnetic resonance imaging

Magnetic Resonance Imaging (MRI) relies on the principles of nuclear magnetic resonance to generate detailed images of the body's internal structures, offering excellent contrast and resolution for tissue characterization. Within the context of sarcopenia assessment, MRI techniques such as T1-weighted imaging, Dixon imaging, T2 mapping, diffusion-weighted imaging (DWI), and magnetic resonance spectroscopy (MRS) provide unique insights into muscle tissue characteristics. In the following subsections the main imaging sequences used in sarcopenia diagnosis and monitoring are recalled and details, while Table 3 summarizes pros and cons of each considered imaging sequence.

**Table 3:** The role of MRI in assessing sarcopenia.

Imaging sequence	Pros	Cons
T1-weighted	1. Excellent contrast for qualitative assessments. 2. Suitable for standard MR scanners and user-friendly. 3. Favorable spatial resolution (0.5 × 0.5 × 3 mm^3^). 4. Quick acquisition time (less than 5 min for a stack of 30 images).	1. Limited utility for other quantitative measurements. 2. Intensity values provide limited information for quantitative assessments. 3. Overestimates muscle mass in cases of fluid accumulation.
Dixon imaging	1. Selective visualization and quantification of fat and water components. 2. Offers “proton density fat fraction” (PDFF) and ‘proton density water fraction’ (PDWF) maps. 3. Proposed as a standardized biomarker for tissue fat concentration.	1. Cannot separate intramyocellular lipid (IMCL) and extramyocellular lipid (EMCL). 2. Inferior spatial resolution compared to T1-weighted images. 3. Potential bias in T1-weighted images, causing non-uniform contrast.
T2 mapping	1. Quantifies tissue changes associated with inflammatory processes. 2. Multi-spin-echo sequences provide comprehensive assessment of T2 relaxation times. 3. Addresses limitations of traditional mono-exponential T2 fitting.	1. Global T2 influenced by dominant fat signal, masking nuanced changes in T2water. 2. Multi-exponential fitting overlooks B1 field inhomogeneities and stimulated echoes. 3. Potential for increased scan times with higher directional accuracy.
DWI/DTI	1. Provides insights into tissue cellularity, integrity, and pathological changes. 2. Fractional Anisotropy (FA) quantifies directional alignment of water molecules. 3. Mean Diffusivity (MD) offers comprehensive assessment of diffusion properties.	1. Anisotropic nature constrained by impermeable barriers in muscle fibers. 2. Increased scan times with higher directional accuracy. 3. Underutilized in muscle imaging, limited data on its applications.
MRS	1. Effectively measures high-energy phosphates (31P-MRS) and glycogen (13C-MRS). 2. 1H-MRS evaluates muscle lipid composition. 3. Provides comprehensive spectral data for various metabolites.	1. Repositioning challenges due to muscle tissue elasticity. 2. Lower precision compared to Dixon imaging, especially in elderly and diseased subjects. 3. Limited applications in muscle imaging, progressively replaced by Dixon imaging and T2 mapping.

### 3.1 T1-Weighted imaging

In the quantification of sarcopenia, T1-weighted imaging plays a crucial role as an MRI sequence. T1-weighted sequences provide excellent contrast between muscle and adipose tissue, forming the foundation for qualitative or semi-quantitative radiological assessments. An appropriate segmentation technique of these sequences is employed for quantitatively measuring the area or volume of tissue compartments. Mainly they are subcutaneous adipose tissue (SAT), defined as the layer of fat located directly beneath the skin but above the underlying muscle tissue (49), intramuscular adipose tissue (IMAT), the presence of adipose tissue within the muscle structure, specifically located between and within muscle fibers (50), or muscle tissue (MT) (51). In comparison to gradient echo sequences, SE sequences demonstrate lower sensitivity to susceptibility artifacts originating from fatty inflation (52). SE T1-weighted sequences can achieve favorable spatial resolution (0.5 × 0.5 × 3 mm^3^), and in extremities, a stack of 30 images can be acquired in less than 5 min (53). While T1-weighted sequences remain standard across all MR scanners and are user-friendly, their intensity values, though valuable for segmentation guidance, offer limited utility for other quantitative measurements. Despite their continued suitability for muscle volume measurement and semi-quantitative assessment of muscle fat infiltration, computed tomography presents a faster alternative less susceptible to motion artifacts (54).

### 3.2 Dixon imaging

Dixon imaging, also known as fat-water separation imaging, is a valuable technique in the quantification of sarcopenia through MRI (53). This method exploits the different resonance frequencies of fat and water protons, allowing for the selective visualization and quantification of these tissue components. Dixon imaging typically involves acquiring multiple image sets at varying echo times, enabling the separation of fat and water signals during post-processing. Dixon imaging employs a variety of sequences, including multi-echo gradient-echo or spin-echo sequences, to acquire images sensitive to both fat and water signals, commonly referred to as proton density fat fraction (PDFF) and proton density water fraction (PDWF) maps. The PDFF is a quantitative MRI parameter used to assess the concentration of fat in a specific tissue or region. It represents the ratio of the density of mobile protons from fat (triglycerides) to the total density of protons from both mobile triglycerides and mobile water in each voxel (55). PDFF is expressed as a percentage and reflects the proportion of the MR signal arising from fat within the imaged tissue. Mathematically, PDFF is defined as: PDFF = (Total density of protons from mobile triglycerides and mobile water/ Density of mobile protons from fat) × 100. PDFF is proposed as the most suitable standardized biomarker of tissue fat concentration, particularly when measured accurately using quantitative MRS or MRI techniques (56), with reported conversions to absolute fat mass in grams (57). The relationship PDFF + PDWF = 1 holds for a given voxel, as the measured MR signal is contributed solely by water and fat protons. However, in the presence of conditions such as edema or fibrosis, PDWF may differ from absolute water content. This underlines the importance of considering potential alterations in water content when interpreting PDFF values, particularly in clinical scenarios involving tissue abnormalities such as edema or fibrotic changes (58). A limitation arises from the inability to separate intramyocellular lipid (IMCL) and extramyocellular lipid (EMCL) in PDFF results. Consequently, PDFF measurements include contributions from IMCL, which are essential for cellular energy metabolism, while the fatty muscle infiltration attributed to EMCL can negatively impact muscle function. While Dixon imaging offers faster acquisition compared to T1-weighted images, its spatial resolution is typically inferior (59). T1-weighted images are often preferred for assessing muscle volume and segmentation tasks, although Dixon images can also serve these purposes (60). A potential drawback of Dixon imaging is the potential for bias in T1-weighted images, resulting in non-uniform contrast across images, a factor mitigated in Dixon images (61).

### 3.3 T2 mapping

In addition to PDFF, T2 relaxation times have gained increasing recognition as a quantitative MRI marker for assessing disease activity in skeletal muscle tissue. This role is particularly relevant in replacing Short Tau Inversion Recovery (STIR) sequences, commonly utilized for semi-quantitative edema assessment (62). Skeletal muscle edema leads to increased T2 relaxation times, serving as an indicator for inflammatory processes that precede the replacement of muscle tissue by adipose tissue. While alterations in T2 are generally non-specific, they can signify the intensity of underlying pathological changes (63). The assessment of T2 in skeletal muscles often involves the use of multi-spin-echo (MSE) sequences, with various fitting approaches proposed to evaluate signal evolution and determine T2 relaxation times (64). The simplest approach involves fitting a mono-exponential signal decay, resulting in an overall “global T2 relaxation time” that encompasses the T2 relaxation times of both water and fat (65). However, in conditions involving fat replacement, this combined T2 is predominantly influenced by the fat signal, masking more nuanced alterations in T2 resulting from other modifications in muscle tissue (66). Consequently, increased intramuscular fat content leads to a higher global T2. This effect is particularly prominent in individuals with elevated intramuscular fat, such as those with neuromuscular disorders. The rise in global T2 due to fat infiltration may obscure subtle pathological changes in T2water, typically of smaller magnitude (65). Various methods have been suggested to selectively assess the T2 of muscle water when there is fat infiltration. These approaches include selectively exciting the water signal or employing conventional fat suppression techniques to suppress the fat signal. However, the efficacy of fat suppression is contingent on precise flip angles and could be compromised in areas with an inhomogeneous transmit (B1+) field (67). An alternative approach involves multi-exponential T2 fitting, a detailed method that allows for a more nuanced analysis of signal decay. This technique goes beyond the simplicity of mono-exponential fitting and provides a comprehensive assessment of T2 relaxation times by considering multiple exponential components within the signal (68). Multi-exponential T2 fitting is particularly advantageous in situations where the conventional mono-exponential approach might oversimplify complex tissue compositions, enabling a more accurate depiction of variations in relaxation times. However, these fitting models often overlook B1 field inhomogeneities and stimulated echoes, affecting T2 values (69). To address issues such as B1 field inhomogeneities and stimulated echoes, an alternative T2 fitting method using extended phase graphs (EPG) has been proposed. EPG simulates signal evolution for various combinations of T2, relative B1, and fat fraction (FF) values, enhancing stability in an inhomogeneous transmit field and correcting for stimulated echoes. This method provides a more robust and accurate assessment of T2 relaxation times in the presence of fat infiltration and other confounding factors (66).

### 3.4 Diffusion weighted imaging

The utility of Diffusion Weighted Imaging (DWI) lies in its ability to assess the movement of water molecules within tissues, providing information on tissue cellularity, integrity, and the presence of pathological changes (70). However, within tissues such as muscle, the presence of impermeable or semi-permeable barriers surrounding muscle fibers and other structures imposes constraints on the diffusion of molecules. This restriction exhibits an anisotropic nature, meaning that the diffusion is directionally dependent (71). Diffusion Tensor Imaging (DTI), as an extension of DWI (72), goes beyond merely measuring the diffusion of water molecules. It specifically assesses the anisotropy of diffusion, providing a more detailed and directional understanding of water molecule movement in biological tissues. The impact of diffusion anisotropy becomes apparent through the assessment of changes in diffusion measurements when altering the direction of gradient pulses (73). Fractional anisotropy (FA) serves as a metric to quantify the directional alignment of water molecules within the tissue, with FA values ranging from 0 to 1. In cases where tissue integrity is maintained, water exhibits movement primarily in a specific direction, resulting in an FA value close to 1. Conversely, in the presence of micro- or macro-structural damages, water molecules assume multiple directions of movement, leading to a decrease in the FA value towards 0 (74). In muscle tissue due to the predominant alignment of water diffusion along the major axis of fibrillary tissues facilitated by their structural arrangement, DTI proves instrumental for fiber tractography. The computation of the diffusion tensor necessitates the acquisition of DWI with high b-values along a minimum of six non-collinear directions, supplemented by a low b-value DWI or a T2-weighted sequence (75). The precision of anisotropy calculation is directly proportional to the number of directions along which diffusion gradients are applied. It is evident that augmenting the number of directions enhances accuracy but comes at the cost of increased scan times. An additional metric derived from the DTI is the mean diffusivity (MD), often interchangeably termed the apparent diffusion coefficient (ADC) (76). Mean diffusivity is a quantitative measure that characterizes the average diffusion of water molecules within a given tissue. MD represents the overall magnitude of diffusion in all directions and is calculated as the average of the three eigenvalues obtained from the diffusion tensor. In the context of DTI, each eigenvalue corresponds to the rate of diffusion along a principal axis within the tissue (77). The mean diffusivity, therefore, provides a comprehensive assessment of the diffusion properties by considering diffusion in both the axial and radial directions (78). DTI remains relatively underutilized in the realm of muscle imaging. However, DTI offers a versatile application in uncovering alterations in muscle diffusivity associated with a spectrum of conditions. One noteworthy instance involves the investigation of changes in muscle diffusivity linked to denervation (79) and metabolic conditions (80). Muscle fiber tears, a common occurrence in various physical activities, also fall within the scope of DTI applications. The technique enables the detection and characterization of muscle fiber tears, contributing to a comprehensive assessment of musculoskeletal injuries (81, 82). Moreover, differences in muscle diffusivity have been explored in the context of distinguishing between osteoporotic and osteoarthritic subjects (83). An area of compelling investigation explores the intricate relationship between muscle properties and external muscle strength, holding profound implications for optimizing training outcomes in athletes and guiding interventions for conditions such as sarcopenia and frailty (84–86). The consistent elevation of FA serves as a notable indicator of inflammatory infiltration and muscle regeneration. These occurrences manifest as transient responses to acute injury and persist as adaptive mechanisms in reaction to the aging process (86).

### 3.5 MRS

MRS has been effectively employed by physiologists for numerous decades, specifically in the study of high-energy phosphates (using 31P-MRS) and glycogen (utilizing 13C-MRS), to measure concentrations of high-energy phosphates or muscular glycogen, respectively (87). Alternatively, 1H-MRS is utilized to evaluate muscle lipid composition. In 1H-MRS, an intensity spectrum is generated based on the relative chemical shifts of lipid metabolites measured in parts per million (ppm) with respect to the universal reference, the methyl 1H signal of tetramethylsilane (TMS), assigned a value of 0 ppm (88). Within this reference system, water protons in living tissues resonate at −4.7 ppm, EMCL at −1.5 ppm, and IMCL at −1.28 ppm. In MR imaging, we use the chemical shift between water and fat to either suppress the fat signal and highlight the water signal or in techniques like Dixon imaging, to separate fat and water signals. However, Dixon imaging struggles to detect the small chemical shift difference (−0.2 ppm) between EMCL and IMCL (89). On the other hand, MRS can distinguish between these lipids, due to structural differences. This requires longer acquisition times, and the MRS signal is typically obtained from a small volume of interest, known as the spectroscopy voxel (about 1 cm^3^) (90). Recent consensus recommendations provide guidance on performing 1H-MRS in skeletal muscle (91). Repositioning the spectroscopic voxel in MRS poses a challenge in muscle imaging due to the elasticity of muscle tissue. This challenge is heightened by the difficulty in reproducible positioning, especially with the high and uneven fat infiltration seen in elderly and diseased individuals. Consequently, MRS exhibits lower precision compared to Dixon imaging (92). In contrast, Dixon imaging is less affected in the liver, an organ frequently imaged using this technique, owing to its more uniform tissue composition. While MRS could potentially overcome these challenges, its applications in muscle imaging are currently limited (93). While 1H-MRS has historically been utilized in muscular dystrophies, it is progressively being supplanted by Dixon imaging and T2 mapping (94). The quantification of IMCLs proves to be a potent tool in exercise and nutrition research, bioenergetics studies, and investigations into insulin resistance (95).

## 4 Bioelectrical impedance analysis (BIA)

BIA is a non-invasive technique that measures the impedance of body tissues to electrical currents to estimate body composition, including muscle mass (96). BIA devices vary in complexity, from handheld devices to multi-frequency analyzers. A systematic review by Aleixo et al. shows that BIA is feasible for evaluating low lean muscle mass for the diagnosis of sarcopenia (97). Two studies demonstrated that BIA yielded comparable results to gold standard methods such as CT and MRI in detecting sarcopenia (98, 99). This evidence has led to the acceptance of BIA as a reliable method in various sarcopenia guidelines, including those in Europe and Asia, as well as the international cachexia consensus (100). However, other authors have indicated that a limitation of BIA in assessing sarcopenia is its reliance on hydration status (101). BIA relies on the assumption that the body is adequately hydrated for accurate measurements of lean body mass. However, variations in hydration levels, such as dehydration or overhydration, can affect the accuracy of BIA results. Additionally, BIA may not provide detailed information about muscle quality or distribution, which could limit its ability to assess sarcopenia comprehensively (102). Moreover, BIA measurements can be influenced by factors such as age, sex, ethnicity, and body composition, which may introduce variability in the interpretation of results (103). Therefore, while BIA is a convenient and non-invasive method for estimating body composition, its limitations should be considered when using it for sarcopenia assessment.

## 5 Emerging technologies

### 5.1 Microwave based diagnostics

Besides the well-known and assessed instrumentations recalled above, an emerging technique which is proposed for various medical diagnostic applications is represented by microwave imaging and sensing. More precisely, studies on the dielectric properties (electric permittivity and conductivity) of human tissues have proved that, in the microwave frequency range, different tissues exhibit different properties, but also differences can be detected in the same tissue according to its physio-pathological status (104–106). This occurrence entails the possibility of exploiting microwave sensing and imaging for diagnostic purposes through the detection and quantification of the electric contrast existing between tissues, made possible by the proper processing of the microwave signal which is transmitted, attenuated and backscattered by the tissues. Microwave imaging has been proposed for several medical applications, ranging from breast cancer diagnosis, brain stroke imaging and monitoring (106–109). Motivated by the evidence that sarcopenia, from a histological viewpoint, entails the substitution of muscle fibers with adipose tissue and given the high electric contrast existing between fat and muscle, recently microwaves have been proposed as a possible non-invasive means to diagnose sarcopenia at an early stage (1, 2, 110). The possibility of exploiting this technique is at a very preliminary phase and, so far, a microwave sensor for the evaluation of muscle quality has been proposed, but no relevant clinical assessment of such a sensor is reported in the literature (111). On the other hand, the great potential of providing 3D images of the examined body area is completely underinvestigated for sarcopenia diagnosis. However, the non-ionizing nature of microwaves and the expected high specificity of the diagnosis make MWI as a very promising tool, for the screening of sarcopenia and, even more, for the monitoring and follow up of the disease.

### 5.2 Near-infrared spectroscopy (NIRS)

Near-Infrared Spectroscopy (NIRS) is an emerging non-invasive method that can be utilized to assess muscle function and possibly sarcopenia. This technique measures the absorption of near-infrared light by the tissues, which can provide insights into tissue composition and blood flow dynamics. NIRS works by emitting near-infrared light into the body's tissues using sensors placed on the skin surface. The light penetrates the tissues and is either absorbed or scattered by the muscles and blood vessels (112). In a preliminary physiological study conducted by Beever et al. involving 26 healthy participants, NIRS proved to be a dependable method for examining skeletal muscle oxidative capacity (113). This result was confirmed by an investigation of Tandirerung et al. in a cohort of 25 young and old non-athletic adult patients (114). NIRS can be particularly pertinent in the study of sarcopenia, where mitochondrial function and muscle metabolism are often compromised due to declining muscle mass and qualitative changes within muscle tissues. A study by Shin et al. demonstrated a high level of agreement between portable NIRS devices and BIA in measuring muscle mass among community-dwelling older adults. This study suggests that NIRS it could be used more widely as a convenient, non-invasive tool for screening and monitoring sarcopenia (115). However, NIRS also has several limitations. The accuracy of NIRS can be affected by factors such as subcutaneous fat thickness, skin color, and tissue heterogeneity. These factors can influence the penetration of near-infrared light into tissues, potentially leading to variations in data accuracy and reliability (116). Another drawback is the limited penetration depth of NIRS, which restricts its use to superficial tissues. This limitation makes it less effective for studying deeper muscles or organs compared to other imaging modalities like MRI or CT. Additionally, NIRS data interpretation can be complex. The signals obtained are influenced by both arterial and venous blood, as well as by the water content in the tissue, which can complicate the distinction between changes in blood flow and actual muscle oxygen consumption (117). Thus, while NIRS offers significant benefits for non-invasive physiological monitoring, its limitations require careful consideration in the design and interpretation of studies or clinical assessments.

## 6 Conclusions

In conclusion, sarcopenia presents a growing global challenge, underscoring the importance of effective diagnostic tools. As summarized in Table 4, various imaging modalities offer valuable insights into muscle quantity and quality, with MRI emerging as particularly promising due to its high-resolution capabilities and fat-water separation techniques. Additionally, ultrasound provides essential information on muscle size and composition, while BIA offers a non-invasive alternative for assessing body composition. The inclusion of NIRS also adds a layer of depth, as it has proven to be a reliable tool in assessing skeletal muscle oxidative capacity, further enhancing our toolkit for diagnosing and monitoring sarcopenia. Standardized protocols are crucial for ensuring consistency in assessments across modalities. Future research should focus on refining these techniques towards a unified framework. Also, the potential of radiomics and artificial intelligence in improving diagnostic accuracy as well as in predicting outcomes in sarcopenia are worthy of consideration in the future research.

**Table 4:** A comparison of the different techniques, highlighting their strengths and limitations in the context of sarcopenia evaluation.

Technique	Advantages	Disadvantages	References
Ultrasound	- Portable - Real-time imaging - No ionizing radiation - Assesses muscle size, architecture, and composition	- Operator-dependent accuracy - Requires standardized protocols - Limited research on sonoelastography and contrast-enhanced US	(21, 25, 30, 46, 47, 48)
Magnetic resonance	- High-resolution - Fat-water separation - Detailed evaluation of muscle quantity and quality	- Expensive - Requires specialized equipment and expertise - Potential challenges with repositioning	(49, 50, 51, 53, 84, 86, 91)
Bioelectrical impedance analysis	- Non-invasive - Estimates body composition including muscle mass - Accepted in various guidelines	- Affected by hydration status - Less detailed on muscle quality or distribution - Influenced by demographic factors	(97, 100, 102, 103)
Microwave imaging	- Non-ionizing radiation - Potential for early-stage detection - High specificity	- Emerging technology - Limited clinical validation - Currently experimental	(1, 2, 110)
Near-infrared spectroscopy	- Non-invasive - Assesses muscle function, oxidative capacity, and blood flow dynamics	- Limited penetration depth - Accuracy affected by subcutaneous fat and skin color - Complex data interpretation	(112, 115–117)
